# Medical Treatment of Lung Cancer: Can Immune Cells Predict the Response? A Systematic Review

**DOI:** 10.3389/fimmu.2020.01036

**Published:** 2020-06-24

**Authors:** Philippe Rochigneux, Alejandro J. Garcia, Brice Chanez, Anne Madroszyk, Daniel Olive, Edward B. Garon

**Affiliations:** ^1^Department of Medical Oncology, Paoli-Calmettes Institute, Marseille, France; ^2^Team Immunity and Cancer, Centre de Recherche en Cancérologie de Marseille (CRCM), INSERM U1068, CNRS UMR 7258, Aix-Marseille Université and Institut Paoli-Calmettes, Marseille, France; ^3^Division of Hematology/Oncology, Department of Medicine, David Geffen School of Medicine at the University of California, Los Angeles, CA, United States; ^4^Cytometry Core Laboratory, David Geffen School of Medicine at the University of California, Los Angeles, CA, United States

**Keywords:** lung cancer, immune biomarker, immune monitoring, chemotherapy, immunotherapy

## Abstract

The landscape for medical treatment of lung cancer has irreversibly changed since the development of immuno-oncology (IO). Yet, while immune checkpoint blockade (ICB) revealed that T lymphocytes play a major role in lung cancer, the precise dynamic of innate and adaptive immune cells induced by anticancer treatments including chemotherapy, targeted therapy, and/or ICB is poorly understood. In lung cancer, studies evaluating specific immune cell populations as predictors of response to medical treatment are scarce, and knowledge is fragmented. Here, we review the different techniques allowing the detection of immune cells in the tumor and blood (multiplex immunohistochemistry and immunofluorescence, RNA-seq, DNA methylation pattern, mass cytometry, functional tests). In addition, we present data that consider different baseline immune cell populations as predictors of response to medical treatments of lung cancer. We also review the potential for assessing dynamic changes in cell populations during treatment as a biomarker. As powerful tools for immune cell detection and data analysis are available, clinicians and researchers could increase understanding of mechanisms of efficacy and resistance in addition to identifying new targets for IO by developing translational studies that decipher the role of different immune cell populations during lung cancer treatments.

## Introduction

Lung cancer is the leading cause of cancer deaths worldwide (1), and systemic treatments (chemotherapy, targeted therapy, or immunotherapy) are required for the vast majority of patients (clinical stages Ib–IV) (2). In the last 5 years, the development of immune checkpoint blockade (ICB) has improved the outcomes of advanced non–small cell lung cancer (NSCLC) (3–5). With this new focus on immuno-oncology (IO), ~200 lung cancer immunotherapy clinical trials are active worldwide (6). The standard initial treatment for advanced NSCLC without driver mutations now includes immunotherapy [monotherapy with high [programmed death ligand 1 (PDL-1) expression or combined with platinum doublet chemotherapy at any level of PDL-1 expression] (7, 8). Despite these treatment strategies, the immune cell dynamic induced by systemic treatments (chemotherapy, targeted therapy, or ICB) is poorly understood. Additional knowledge about this immune dynamic could be important to better understand the mechanisms of both efficacy and resistance to anticancer drugs. Further, it is possible that this exploration could identify new targets for immunotherapy. Compared to the field of melanoma (9), data are limited and very fragmented among lung cancer studies. Consequently, we conducted a systematic review of lung cancer studies that look at immune cell populations (Box 1). First, we will describe techniques that allow immune cells detection, focusing on recent discoveries. Second, we will review the immune predictors of response to medical treatments in lung cancer, studied at baseline or during treatment.

Box 1 **Method: search strategy.**

Database: PubMed, Google Scholar, Scopus, Web of Science, ASCO abstracts, ESMO abstracts, WCLC abstracts

Database key words (MeSH terms and title/abstract) = “immune cells” OR “immune biomarker” OR “immune monitoring” OR “immuno-monitoring AND “lung cancer” OR “lung neoplasm” OR “non–small cell lung cancer” OR “small cell lung cancer”

Time limit: last 10 years (2009–2019)

Language: English only

Species: Human only

Inclusion criteria: Lung cancer treated with chemotherapy (CT), targeted therapy, or immunotherapy (IT), in an adjuvant setting, locally advanced or metastatic setting. Data available for immune cell population (e.g., CD3 T cells) or immune cell markers (e.g., PDL-1 expression in macrophage or dendritic cells).

## Classical and New Technics of Immune Monitoring

### New Developments in Immunohistochemistry and Immunofluorescence

**Figure 1:**
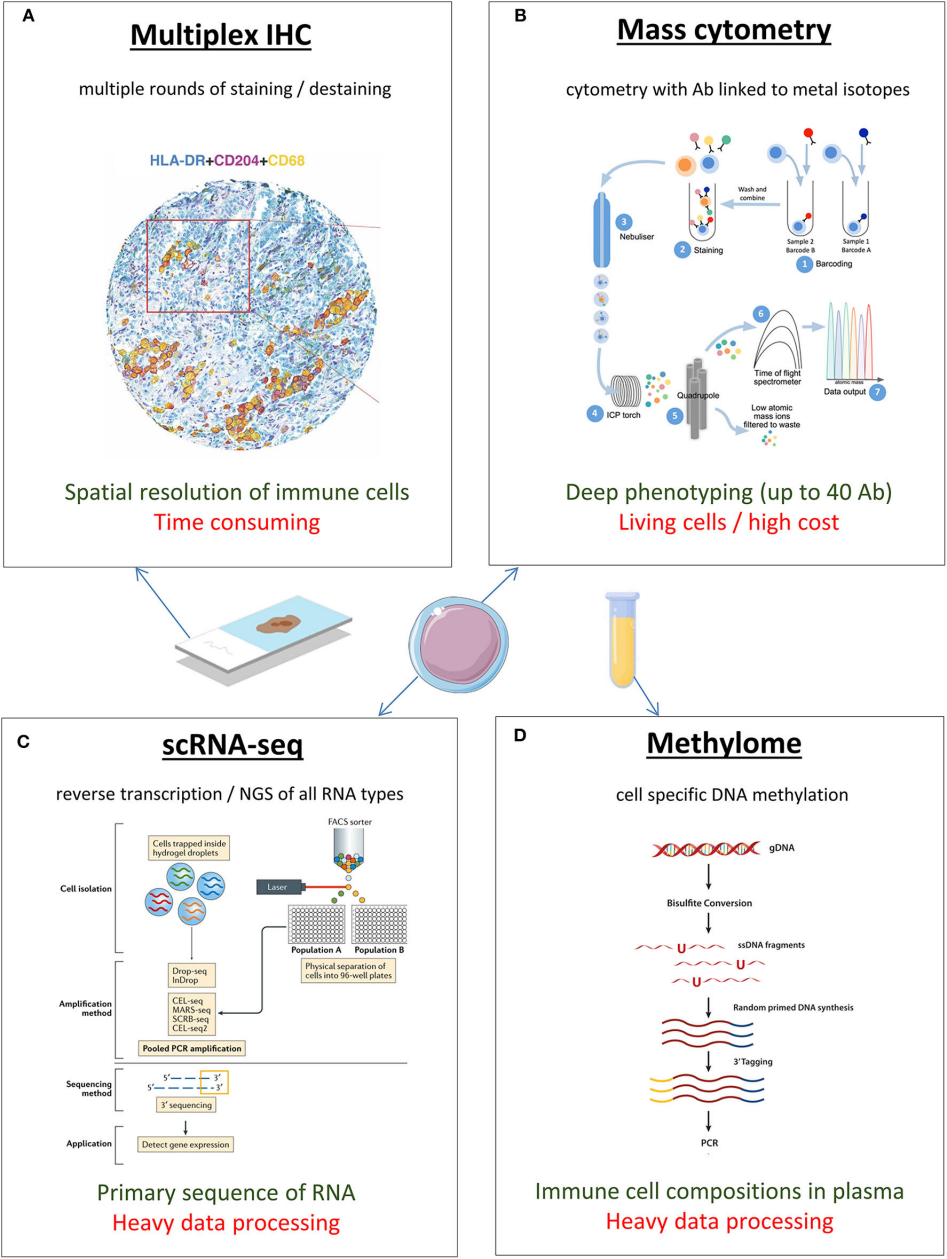
New techniques for immune monitoring, in blood or tumor microenvironment of lung cancer. Colors in the text (bellow each image): green is for the main advantage; red is for the main disadvantage. Illustrations are adapted from the following references: **(A)** Rakaee M et al. (11); **(B)** Stern et al. (12); **(C)** Papalexi et al. (13); **(D)** Epicentral.com. Ab, antibody; IHC, immunohistochemistry; NGS, next-generation sequencing; scRNA-seq, single-cell RNA sequencing.

Since its invention by Albert Coons in the 1940s, immunohistochemistry (IHC) has been the gold standard in studying immune cell infiltration inside the tumor and the tumor immune microenvironment (TIME) (10) (Figure 1). Briefly, the classical IHC is the staining of formalin-fixed, paraffin-embedded (FFPE) tissues with antibodies linked to an enzyme or fluorescent dye (14). This approach allows preservation of the tumor and TIME architecture, allowing spatial resolution. However, the main inconvenience of classical IHC is that a maximum of 2 antibodies are stained on one slide, necessitating a lot of tumor material and complicating the study of multiple cell populations. “Multiplex IHC” is a new technique allowing the visualization and quantification of specific immune cell populations by using multiple markers to identify different subsets [e.g., subset of dendritic cells (DCs) or CD8^+^ T cells] (15, 16). Multiplex IHC allows consecutive staining on a single slide (up to 10 antibodies) by using multiple rounds of staining and destaining (15). Several commercial solutions are available, mainly based on proprietary fluorescent probes and frozen materials (16). A nonproprietary assay called MICSSS (multiplexed immunohistochemical consecutive staining on single slide) has been developed based on chromogens and virtual color assignment (17). For example, in NSCLC patients (*n* = 75), MICSSS allowed staining of T cells, regulatory T cells (T_Reg_), B cells, DCs, macrophages, and neutrophils together on a single slide, allowing for meaningful co-localizations (17). To improve the semiquantitative aspect of IHC, several algorithms have been developed to automatically analyze the slides of FFPE tumors (e.g., AQUA^®^), both for immunofluorescence (IF) and IHC, with an increasing use in translational research (18, 19). In the context of immune checkpoint inhibitors, quantitative IF and AQUA^®^ were used to determine a “dormant” tumor-infiltrating lymphocyte (TIL) signature (elevated TILs with low activation and proliferation) associated with survival benefit (20). Recently, the performance of several biomarkers of anti–PDL-1 was studied in a meta-analysis of 45 studies; multiplex IHC/IF was associated with improved performance over PDL-1 IHC, tumor mutational burden, or gene expression (21). Finally, a new technique called “imaging mass cytometry” couples the principles of IHC and mass cytometry; tissue sections are stained with antibodies (up to 40) linked to rare metal isotopes, and an ultraviolet laser ablates the material spot by spot, which is then sent to the CyTOF (cytometry by time-of-flight) mass detector (see below cytometry section) (22).

### Immune Gene Expression Profiling

As cell types have distinct transcriptional profiles, it is possible to define immune cell populations by gene expression analysis. Traditional techniques, such as microarrays or NanoString nCounter^®^ systems already, allow this identification, but the development of RNA-seq has transformed gene expression analyses into a powerful tool to identify cell populations. The main advantage of RNA-seq is to determine the primary sequence and relative abundance of each RNA molecule without previous knowledge of the sequence (using retrotranscription to cDNA and next-generation sequencing) (23). Furthermore, with microfluidics and barcodes (24), samples can be analyzed on a single-cell basis (single cell (sc) RNA-seq), allowing precise characterization of cell types in samples with cellular heterogeneity, such as lung (25–27). Sc RNA-seq also has the potential to define novel cell subtypes in blood (28) or solid tissues (29) and to follow cell differentiation with RNA velocity (30). Of course, RNA-seq requires important computational statistical analysis, but machine learning methods such as Cibersort, XCell, and MetaNeighbor have been recently developed to simplify the characterization of cell composition from transcriptome data (31–33).

### Methylation Patterns

Epigenetic modifications, particularly DNA methylation, are crucial biological processes, allowing for the expression of specific cellular phenotypes from a common genetic background (34). DNA methylation is the addition of a methyl group to the C5 carbon residue of cytosines by DNA methyltransferases. Interestingly, these methylation patterns are cell type specific, and several studies describe that the methylome distinguishes cell lineages with high sensitivity and specificity (35–37). Technically, methylome analysis begins with a bisulfite conversion, as sodium bisulfite converts cytosines into uracils, whereas methylcytosines remain unmodified (38, 39). Subsequent amplification gives rise to two polymerase chain reaction products that are sequenced. From there, the sequences are aligned to a reference sequence, which can prove to be challenging (40, 41). As the tools for DNA methylation mapping are improving, and the required amount of DNA is decreasing, DNA methylome can now predict cell compositions in plasma (42). Consequently, plasmatic immuno-monitoring studies could be published in the future.

### Cytometry (Flow and Mass)

Invented in the 1960's, flow cytometry is a technique that studies the properties of a single cell in a liquid environment using fluorophore-linked antibodies (43). Lasers excite the fluorophore-linked antibodies at a certain spectrum, and the detectors record the emission spectrum (44). This signal is proportional to the expression of intracellular or extracellular cell markers, allowing for the identification of cell types–based markers of interest (45). Flow cytometry has quickly become a routine technique not only in malignant hematology and infectious disease studies, but also in drug development and drug monitoring (45–47). Successive technical improvements have broadened the number of excitation lasers (up to 10; BD InFlux) and the available fluorophores (notably with tandem and brilliant violet) allowing classical 18+ antibody panels. However, the multiplication of antibodies in a limited wavelength range (350–550 nm) leads to spectral overlap of fluorophores, requiring complex and time-consuming compensation setup before analyzing the cytometry data (48).

Around 2010, DVS Sciences Company and the Nolan Lab at Stanford University developed mass cytometry (CyTOF), a technique using antibodies tagged with rare earth metal isotopes (lanthanide series of the periodic table) (49). After staining with antibodies, the cells are nebulized and ionized with an argon inductively coupled plasma, and the ratio charge/mass is used to get a specific time of flight (TOF) proportional to the marker of interest (50). Thanks to the ability to distinguish isotopes clearly, up to 40 parameters can be studied in a single cell simultaneously, which is very useful for precious samples (51). Comparative analysis with flow cytometry showed that mass cytometry had a strong accuracy and reproducibility (52). The development of CyTOF and its very large amount of biological data initiated a new era of high dimensional analyses (53). Several algorithms were created (Spade, viSNE, Citrus) to automatically cluster cell populations and provide a global map of cell signaling responses to interventions (54–57). Use of CyTOF led to several interesting results in predictors of response to ICB in melanoma (58, 59). Although the cost of the instrument is high, and the amount of the data generated requires some statistical expertise, the value of mass cytometry as a tool for immune monitoring is quickly increasing.

### Functional Tests

While understanding the cellular composition of TIME is important, knowing the functional status of the infiltrating immune cells tremendously enhances the value of the data. Researchers can study the proliferation of lymphocytes [a prognostic marker in several tumor types (60, 61)], thanks to the incorporation of ^3^H thymidine after stimulation with mitogens (62, 63). Cytotoxicity properties of CD8^+^ T cells or natural killer (NK) cells are also frequently measured with radioactive chromium (^51^Cr) assays in which target cells are loaded *in vitro* with radioactive chromium, and lysis is determined by measuring chromium released by dying cells in the supernatant (64, 65). Developed in 1968, this technique is still the gold standard for evaluation of cytotoxicity but requires handling radioactivity and autologous tumor targets. Interestingly, nonradioactive tests for cytotoxicity are emerging, based on flow cytometry (66) or microscopy of fluorescent target cells (67). Another classical way to measure T cell and NK cell cytotoxicity is enzyme-linked immunospot (ELISPOT), a sensitive immunoassay that measures the frequency of cytokine-secreting cells at the single-cell level (68, 69). As cytotoxic effector cells, on the one hand, will induce killing by different mechanisms (perforin, granzyme, Trail) but at the same time produce cytokines important for immune and inflammatory functions [such as interferon (IFN) and tumor necrosis factor (TNF)], IFN-γ ELISPOT is often used as a surrogate marker for cytotoxic properties of effector T cells. Other ELISPOT analyses also include granzyme B or perforin, two secreted proteins involved in perforation and caspase activation of the target cells (70). Lastly, measuring cytokine production with ELISPOT or intracellular flow cytometry allows assessment of the activation of CD4^+^ T cells (IL-2 production) or differentiation of subpopulations, such as CD4^+^ T_H_1 (IFN-γ, IL-2, IL-6, IL-12, IL-21, and TNF-α), T_H_2 (IL-4, IL-5, and IL-13), and T_H_17 (IL-17) cells.

## Baseline Predictors of Response

### Immune Predictors of Chemotherapy and Targeted Therapy Response

**Figure 2:**
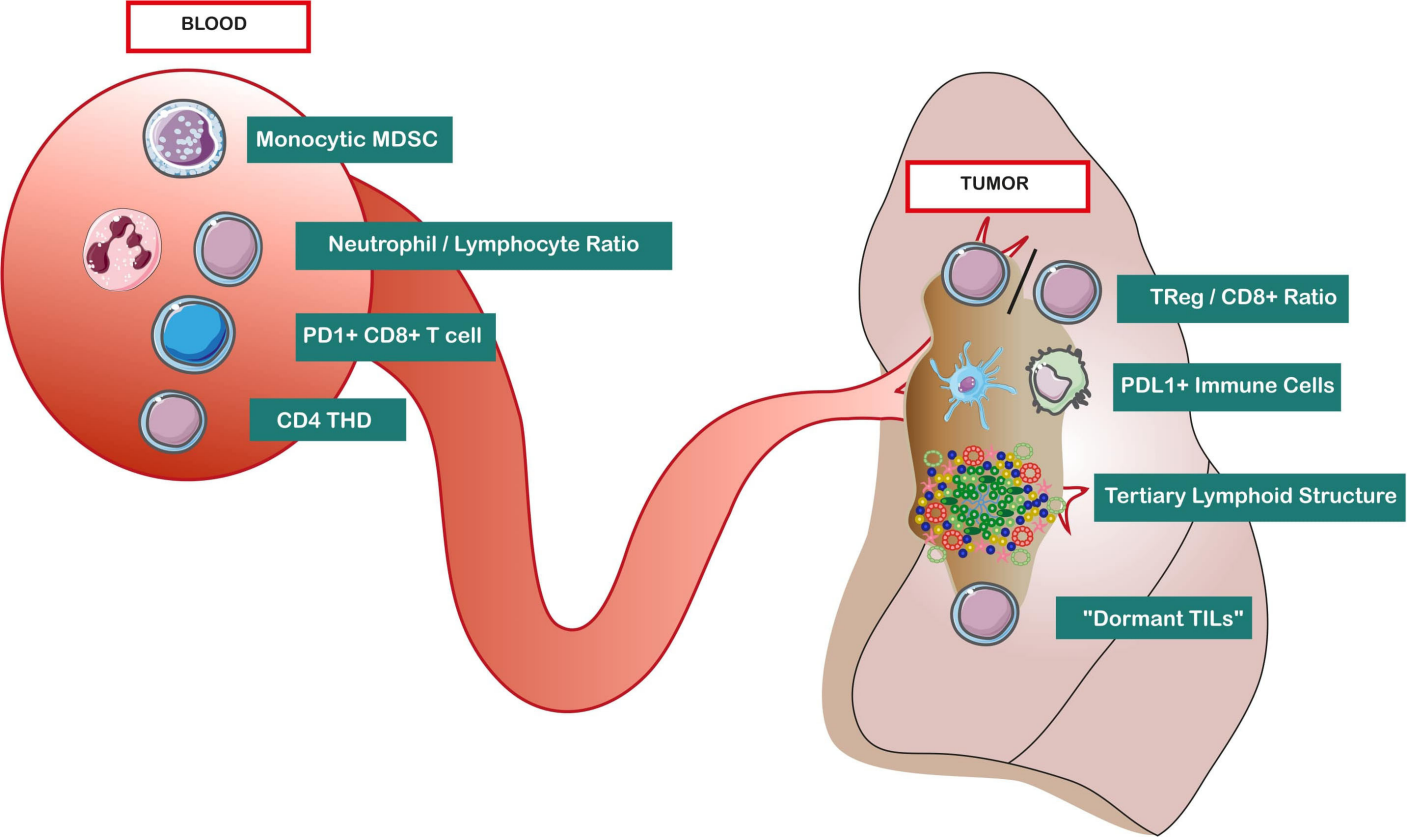
Baseline immune predictors of response to lung cancer medical treatments, in blood and tumor microenvironment. “Dormant TIL” is defined as CD3^high^ granzyme B^low^ Ki-67^low^. Abbreviations: CD4 THD, highly differentiated CD4+ T cells (CD27– CD28^low/−^); Mo-MDSC, monocytic myeloid-derived suppressor cells; PDL-1, programmed death ligand 1; TIME, tumor immune microenvironment; T_Reg_, regulatory T cells.

The majority of studied biomarkers for efficacy of platinum doublets are not immune biomarkers but tumor biomarkers, studied at a genomic (71), transcriptomic (72), or protein level (73) (Figure 2). However, a few studies have raised the question of TILs in the context of chemotherapy. For example, in an analysis of 1,586 resected lung cancers treated with platinum-based adjuvant chemotherapy, an intense infiltration of TIL in IHC was a positive prognostic marker, but had no predictive value for efficacy of the platinum doublets (74). In a metastatic setting, of 159 patients analyzed for TILs based on IHC, none of the T-cell subsets alone (CD8, CD4, T_Reg_) were associated with tumor response, but a low T_Reg_/CD8^+^ ratio was associated with more tumor response to platinum doublets in multivariate analysis [odds ratio = 4.17, 95% confidence interval (CI) = 1.02–13.37, *p* = 0.029] (75). Finally, even if less studied, B cells can organize in the stroma with T cells and DCs into tertiary lymphoid structures (TLSs) that are ectopic lymphoid organs at site of inflammation (76). In 122 NSCLCs treated with neoadjuvant chemotherapy and surgery, density of follicular B cells and DCs organized in TLS was associated with an improved disease-specific survival (DSS) after 50 months' follow-up (median DSS for B cells ^Hi^/DCs ^Hi^ >60 vs. 21 months for B cells ^Low^/DCs ^Low^, *p* = 0.007) (77).

Among immune cells biomarkers in the blood, one of the most studied is monocytic myeloid-derived suppressor cells (Mo-MDSCs: CD33^+^, HLADR^−^, CD11b^+^, CD14^+^), an immature myeloid cell population inhibiting proliferation and cytotoxicity of T cells. In a cohort of 24 stage IV patients treated with chemotherapy (cisplatin/pemetrexed), progressors had higher rates of a subset of Mo-MDSCs, CD11b^+^, CD14^+^, and S100A9^+^ (damage-associated molecular pattern molecules). Patients with a decreased frequency of these cells under the median had significantly longer progression-free survival (PFS) (9.2 vs. 3 months, *p* < 0.001) (78). In another study of 60 patients with advanced NSCLC treated with chemotherapy, progressive disease was associated with more baseline Mo-MDSCs (HLA-DR^−/low^, CD14^+^), and a baseline number of Mo-MDSCs under the median was also associated with longer median PFS (9 vs. 3 months, *p* < 0.001) (79). This effect of Mo-MDSCs on PFS was consistent both in frequency (%) and absolute number (cells/μL). Another interesting blood biomarker is the pretreatment neutrophil-to-lymphocyte ratio (NLR). In 182 stage IV patients treated with platinum doublets (80), a high NLR (>2.63) was associated with worse PFS [hazard ratio (HR) = 1.81, *p* = 0.018] and overall survival (OS) (HR = 1.76, *p* = 0.02) in multivariate analysis, suggesting an independent detrimental effect of inflammation in response to chemotherapy. Next to NLR, a 2019 abstract suggests that high prechemotherapy absolute lymphocyte count is associated with favorable outcome in stage IB–III NSCLC patients who received adjuvant chemotherapy after surgical resection (81).

In the field of targeted therapy (e.g., EGFR and ALK inhibitors), data about baseline immune biomarkers are very scarce. Similar to chemotherapy, a retrospective analysis of 152 stage III/IV patients treated with EGFR tyrosine kinase inhibitors (TKIs) also found that a high NLR (>2.11) was an independent prognostic factor for longer OS (HR = 1.07, *p* = 0.03) (82). Additionally, in a prospective cohort of 33 patients treated with EGFR-TKI (mainly erlotinib) (83), flow cytometry analyses on peripheral blood mononuclear cells reported that a high baseline PDL-1^+^ CD3^+^ T cells predicted shorter OS in multivariate analysis (HR = 3.52, 95% CI = 1.09–11.4, *p* = 0.036).

### Immune Predictors of Checkpoint Inhibitors Response

In the TIME, several baseline biomarkers are associated with response to checkpoint inhibitors, which we can schematically resume in 2 categories: (1) immune infiltrate or immune exclusion and (2) immune function or immune dysfunction of TIL infiltrate.

First, the pattern of immune infiltration (immune infiltrate vs. immune exclusion) seems crucial, as pejorative outcomes were described for lung tumors without immune CD8^+^ infiltration (immunological ignorance) or with immune CD8^+^ infiltrate outside of the tumor (excluded infiltrate) (84, 85). However, compared to melanoma, few data about immune exclusion are available in the ICB area in lung cancer (86). Recently, in 39 NSCLC patients treated with diverse ICB, the level of T-cell infiltration (CD3^+^) assessed by quantitative multiplex IF was 2.4-fold higher in patients with durable clinical benefit (*n* = 16) (20).

Second, data suggest that the presence of infiltration *per se* is not predictive, but depends of the precise nature of TIL infiltration and TIL functional state. Indeed, in the previous study, the highest response to ICB was observed in a specific subgroup called “dormant TIL signature” (CD3 ^high^ granzyme B ^low^ Ki-67 ^low^, *n* = 7) (20). Interestingly, this signature was independent of PDL-1 expression in tumor cells or tumor mutational burden. Similarly, in the phase 1 study of atezolizumab in NSCLC patients (*n* = 53), best responses were observed in a specific subgroup of tumor-infiltrating immune cells with PDL-1 expression (macrophages, DCs, and lymphoid cells) (84). The presence of this immune infiltrate correlated with an improved objective response rate (ORR) (83% of ORR in the subgroup of immune PDL-1 expression >10%, compared to 14%−20% of ORR otherwise). Moreover, this association with atezolizumab response was stronger for PDL-1 expression in tumor-infiltrating immune cells than for PDL-1 expression in tumor cells. These data suggest that PDL-1 has a major role in pretreatment immunosuppression that can be reversed by that checkpoint inhibitor. Gene expression profile can also assess the functional state of TILs: in a prospective setting, IMpower150 study of PDL-1 inhibitor atezolizumab used the biomarker CD8^+^ Teff gene signature, defined by PDL-1, CXCL9, and IFN-γ mRNA expression from baseline tumor tissue (87). The benefit of the combination treatment atezolizumab, bevacizumab, carboplatin, paclitaxel (ABCP) vs. bevacizumab, carboplatin, paclitaxel (BCP) was particularly important in the Teff-high subgroup (median PFS ABCP = 11.3 months vs. median PFS BCP = 6.8 months) compared to the general population (median PFS ABCP = 8.3 months vs. median PFS BCP = 6.8 months). If confirmed, Teff mRNA signature is a promising surrogate of exhausted peritumoral T cells that can be reversed by ICB (85). Interestingly, this transcriptomic IFN-γ signature was independent of PDL-1 tumor expression (IHC). Increasing IFN-γ response is a current goal of pharmacological development to boost ICB, notably with STING (stimulator of interferon genes) agonist (88). Finally, there are limited data for alternative checkpoints such as LAG-3 expression on T cells, associated with shorter OS with PD-1 inhibitors (89). In conclusion, an approach combining both T cell exclusion and dysfunction [as the gene expression TIDE computational method (85)] may be particularly relevant to predict ICB response.

In the blood, contrary to the field of melanoma where several baseline immune predictors of response are described [e.g., baseline CD14^+^ CD16^−^ HLA-DR^hi^ monocytes (58) or baseline Ki67^+^ PD-1^+^ CD8^+^ T cells (59)], only one large study is available in NSCLC, including 466 patients treated with diverse PD-1/PDL-1 inhibitors. The authors studied the impact of baseline Lung Immune Prognostic Index (LIPI), combining derived neutrophils/(leukocytes minus neutrophils) ratio (dNLR) and Lactate dehydrogenase (LDH) (90). Poor baseline LIPI, combining dNLR greater than 3 and LDH greater than upper limit of normal, was correlated with worse outcomes for ICB treatment in patients with NSCLC, but not with chemotherapy. Median OS was 4.8 (95% CI, 3.6–7.7) vs. 10.0 (95% CI, 7.3–12.6) vs. 16.5 (95% CI, 11.4–34.0) months for the poor, intermediate, and good LIPI groups, respectively. Similarly to chemotherapy (80, 82), these data raise the important question of the detrimental effect of baseline inflammation for ICB treatment in NSCLC. Recently, our group presented mass cytometry analysis about baseline predictors of pembrolizumab efficacy in NSCLC on KEYNOTE-001 using machine-learning algorithm (91). Three predictors of response were identified (classical monocytes perforin^+^ granzyme^+^/central memory CD4^+^ T cells ICOS^+^ CD28^+^ PD1^+^/41BB^+^ and perforin^+^ effector CD8+ T cells), and prospective validation is ongoing. Interestingly, the positive impact of classical monocyte in baseline has been previously published in melanoma (58).

Recently, the importance of myeloid cells is emerging in lung cancer; in 32 NSCLC patients treated with ICB (pembrolizumab, nivolumab, atezolizumab), a high proportion of myeloid cells expressing PDL-1 (PDL-1^+^ CD11b^+^ myeloid cells) was associated with objective response (92). Additionally, a functional CD4 immunity also seems important; among 51 NSCLC patients treated with anti–PDL-1, those with an increased proportion of highly differentiated CD4 (T_HD_: CD27^−^ CD28^low/−^) had improved PFS/OS (93). The majority of these CD4^+^ T_HD_ cells corresponded to nonsenescent, nonexhausted memory CD4 cells.

Finally, some data are emerging about the worrying phenomenon of hyperprogression; in 263 NSCLC patients treated with PD-1/PDL-1 inhibitors, a lower frequency of effector/memory CD8^+^ T cells (CCR7^−^ CD45RA^−^) and a higher frequency of severely exhausted populations (TIGIT^+^ T cells among PD-1^+^ CD8^+^ T cells) were associated with hyperprogression (n = 55/263) and inferior survival rate (94).

## Dynamic Predictors of Response

**Figure 3:**
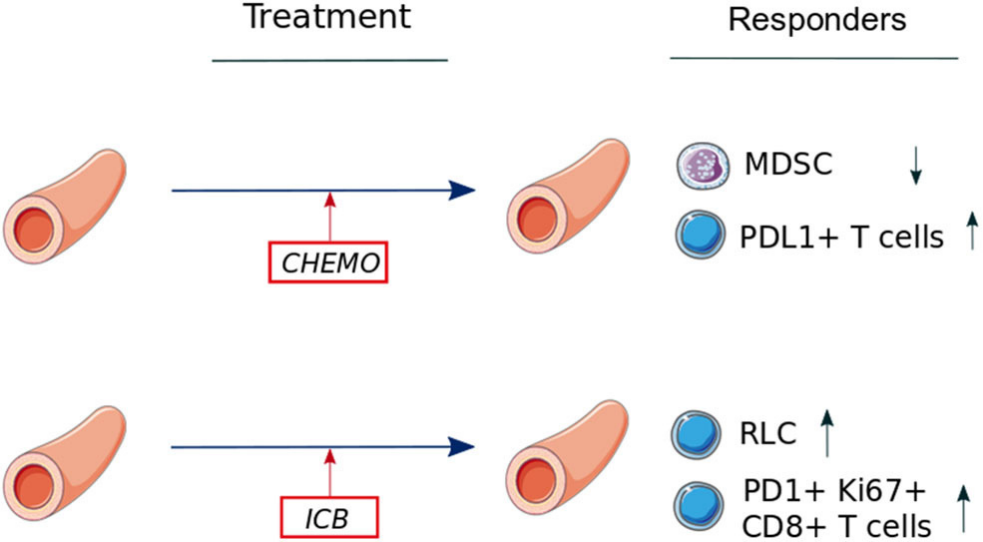
Dynamic changes in immune cell populations during chemotherapy or immune checkpoint blockade in lung cancer (data available in blood only). Abbreviations: Chemo, chemotherapy; ICB, immune checkpoint blockade; MDSC, myeloid-derived suppressor cells; PD-1, programmed death 1; PDL-1, programmed death ligand 1; RLC, relative lymphocyte count.

Baseline predictive biomarkers may be sufficient to identify patients benefiting from medical treatments (Figure 3). However, dynamic biomarkers have theoretical added value. Immune system is a highly dynamic system with many switches, thresholds, and feedforward and feedback loops (95). Consequently, immunity is very sensitive to initial conditions, and minuscule differences may go undetected. Moreover, immune system has been described as a complex system with randomness and stochastic variations (e.g., lymphocyte fate decision) (96), where small differences can lead to massive consequences downstream (minor bacterial exposure leading to a septic shock, targeting one single molecule leading to cytokines storm). If dynamic biomarkers are just emerging, they are promising tools to identify secondary mechanisms of resistance, especially if the selected timepoints are chosen wisely (at the time of progression or response). Finally, understanding these resistance mechanisms could help develop new IO combination therapies (97).

### Immune Predictors of Chemotherapy and Targeted Therapy Response

Similar to baseline markers, one of the most studied dynamic immune cells blood biomarker is MDSCs (CD33^+^ CD11b^+^). Liu et al. (98) reported variation of granulocytic MDSCs (CD33^+^ CD11b^+^ CD14^−^ CD15^+^) in advanced NSCLC patients treated with chemotherapy; nonprogressors (partial response or stable disease, *n* = 41) decreased their Gr-MDSC frequency compared to baseline (*p* < 0.0001), contrary to progressors (*n* = 37), who kept similar frequency. The authors also described a negative correlation between Gr-MDSC frequency and CD8^+^ T cells in blood, consistent with the known immunosuppressive effect of MDSCs on T cells. Although less convincing statistically, another study of 46 patients with unresectable NSCLC, treated with platinum-based chemotherapy, reported that disease progression was associated with significantly higher levels of MDSC subpopulations (CD15^+^ and CD14^+^) compared to patients with disease control (100).

Except limited data about cytokines variations (101), the only immune variations described during EGFR inhibition are those described in the previously cited study about PDL-1^+^ CD3^+^ T cells (83). A value over the median after 1 week of EGFR inhibitors is associated with a decreased OS (HR = 6.49, 95% CI = 1.9–21.8, *p* = 0.002). These findings may reflect an immune resistance mechanism occurring in the PD-1/PDL-1 pathway after initiation of the targeted therapy (83).

### Immune Predictors of Checkpoint Inhibitors Response

Unfortunately, despite several studies describing the evolution of systemic immunity during checkpoint inhibitors in melanoma [e.g., increase of central memory CD4^+^ T cells (102)], there are few data available for lung cancer patients. In the previously cited phase 1 study of atezolizumab, an increase of CD8^+^ HLA-DR^+^ Ki67^+^ T cells in blood was seen at C2D1 (second infusion), but this variation was not correlated with atezolizumab response (84). Similarly, after 6 weeks of treatment, durvalumab significantly increased tumor gene expression of T cell chemotactic chemokine CXCL9, the checkpoint molecule LAG3, and IFN-γ, but the clinical effects of these immune variations was unclear (103).

However, some positive results are emerging; in an American Society of Clinical Oncology 2019 abstract, in 88 NSCLC patients who received anti-PD1 therapy in a single institution, NLR and relative lymphocyte count (RLC) were recorded at baseline and during the treatment (104). Using median RLC at 4 weeks as a threshold, patients with high RLC at 4 weeks had significantly favorable survival (log-rank *p* < 0.0001). For patients with acquired resistance to therapy, RLC increased early during treatment followed by a decrease at the time of progression. Moreover, it is known that PD-1 inhibitors can rescue exhausted T cells (59). Interestingly, in a cohort of 29 NSCLC (responders: *n* = 10) treated with three different anti–PD-(L)1 agents (pembrolizumab, nivolumab, and atezolizumab), 80% of NSCLC patients with partial clinical responses presented early proliferative CD8^+^ T cells, which were both PD-1^+^ (exhausted) and Ki67^+^ (in replication) (99). Patients with an early proliferation of these PD-1^+^ Ki67^+^ CD8^+^ T cells in the blood within 4 weeks of treatment initiation had higher response rates (99). In another study in NSCLC patients (*n* = 13), the same team described that PD-1^+^ CD8^+^ T cells activated by PD-1 therapy were mostly CD28^+^, suggesting a potential role for this costimulation molecule in ICB response (105). Altogether, these studies describe a positive outcome of an early PD-1^+^ CD8^+^ T-cell blood response unleashed by blockade of the PD-1 pathway.

## Concluding Remarks

To be concise, we voluntarily limited this review to medical treatments of lung cancer and immune cells, even if some interesting immunological data are emerging about neoantigens or T Cell Receptor (TCR) repertoire (106). Inside this field, baseline data showed that a low number of Mo-MDSCs, a low NLR, and a high number of TLS improve platinum-based chemotherapy outcomes. Dynamic data showed that the decrease of MDSCs and the increase of PD1^+^ CD8^+^ T cells improve chemotherapy or immune checkpoint inhibitors outcomes (Table 1). However, the knowledge about immune dynamics induced by medical treatments is still scarce, and none of these markers have sufficient level of evidence to be used in clinical practice. Academic research needs to lead in designing translational studies capable of rigorously evaluating this kind of data. Deciphering immune dynamics induced by medical treatments would aid in understanding the mechanisms of resistance and could also identify new targets for immunotherapy. Powerful and robust tools such as multiplex IHC/IF, RNA-seq, methylome, or mass cytometry are now available, together with software for data analysis. In the future, multi-omics approach will help integrate the different data about immune cells biomarkers (107). In that perspective, systems cancer immunology may soon help guide clinical decision-making (108). Lung cancer physicians and researchers should seize this opportunity to pursue immune monitoring together with drug development in IO.

**Table 1:** Immune cell populations predicting efficacy in lung cancer medical treatments (including cells function and references).

**Immune biomarker**	**Definition**	**Location**	**Outcome**	**References**
Treg/ CD8^+^ ratio	CD4^+^ FoxP3^+^	Tumor	↑ Chemo response rate	(75)
Tertiary Lymphoid Structures	B cells, DCs, CD4^+^ CD8^+^ T cells	Tumor	↑ Chemo PFS	(77)
“T_eff_” lymphocytes signature	PD-L1, CXCL9, and IFNγ mRNA	Tumor	↑ ICB PFS/OS (signature]	(87)
“Dormant” lymphocytes	CD3^+^ Granzyme B^−^ Ki67^−^	Tumor	↑ ICB response rate	(20)
CD4^+^ highly differentiated	CD27^−^ CD28^low/−^	Blood	↑ ICB response rate	(93)
PDL1^+^ immune cells	PDL1^+^ macro, DCs, lymphocytes	Tum/Blood	↑ ICB response rate	(83, 84, 92)
“Reactivated” lymphocytes	CD8^+^ PD1^+^ Ki67^+^	Blood	↑ ICB response rate	(99)
Neutrophil lymphocyte ratio (NLR]	Neutrophil/Lymphocyte	Blood	↓ Chemo PFS/OS (high NLR]	(80)
Lung immune prognostic index (LIPI]	dNLR >3; LDH > ULN	Blood	↓ ICB PFS/OS (high LIPI]	(90)
Myeloid derived suppressor cells	CD33^+^ HLADR^−^ CD11b^+^	Blood	↓ Chemo/ICB response rate	(78, 98)

*Data are presented for published studies (not for abstract only). Immune populations with a positive or detrimental outcome are highlighted in green or red, respectively. CXCL9, chemokine (C-X-C motif) ligand 9; DCs, dendritic cells; IFN, interferon; Chemo, chemotherapy; ICB, immune checkpoint blockade; NLR, neutrophil-to-lymphocyte ratio; PD-1, programmed death 1; PDL-1, programmed death ligand 1; PFS, progression-free survival; OS, overall survival, w, weeks*.

## Author Contributions

PR, DO, and EG designed the review. AG helped with the methodological and technical aspects. BC and AM helped with designing the figures and improving medical relevance. PR and EG wrote the paper with input from all authors. All authors contributed to the article and approved the submitted version.

## Conflict of Interest

EG reports funds to his institution from AstraZeneca, Boehringer Ingelheim, Bristol Myers Squibb, Eli Lilly, Genentech, Mirati, Merck, and Novartis. DO was co-founder of Imcheck Therapeutics and Emergence Therapeutics and has research funds from Imcheck Therapeutics, Alderaan, BMS, Talix, Cellectis and Emergence Therapeutics. The remaining authors declare that the research was conducted in the absence of any commercial or financial relationships that could be construed as a potential conflict of interest.

## Abbreviations

CyTOF: cytometry by time-of-flight
DCs: dendritic cells
ELISPOT: enzyme-linked immunospot
FFPE, formalin-fixed: paraffin-embedded
HR: hazard ratio
ICB: immune checkpoint blockade
IF: immunofluorescence
IFN: interferon
IHC: immunohistochemistry
IO: immuno-oncology
LAG3: lymphocyte-activation gene 3
MDSC: myeloid-derived suppressor cells
Mo-MDSC: monocytic myeloid-derived suppressor cells
NGS: next-generation sequencing
NLR: neutrophil-to-lymphocyte ratio
NSCLC: non–small cell lung cancer
OS: overall survival
OR: odds ratio
PBMC: peripheral blood mononuclear cell
PD1: programmed death 1
PDL-1: programmed death ligand 1
PFS: progression-free survival
PR: partial response
RNA-seq: RNA sequencing
SD: stable disease
STING: stimulator of interferon genes
TIGIT: T cell immunoreceptor with Ig and ITIM domains
TIL: tumor-infiltrating lymphocytes
TIME: tumor immune microenvironment
TKI: tyrosine kinase inhibitors
TLS: tertiary lymphoid structures
TMB: tumor mutational burden
TReg: regulatory T cells.
